# Aging and the Division of Labor of Theory of Mind Skills in Metaphor Comprehension

**DOI:** 10.1111/tops.12785

**Published:** 2025-02-17

**Authors:** Irene Ceccato, Serena Lecce, Luca Bischetti, Veronica Mangiaterra, Chiara Barattieri di San Pietro, Elena Cavallini, Valentina Bambini

**Affiliations:** ^1^ Department of Psychology “G. D'Annunzio” University of Chieti‐Pescara; ^2^ Department of Brain and Behavioral Sciences University of Pavia; ^3^ Laboratory of Neurolinguistics and Experimental Pragmatics (NEPLab), Department of Humanities and Life Sciences University School for Advanced Studies IUSS Pavia

**Keywords:** Pragmatics, Metaphor, Theory of Mind, Aging, Figurative language, Experimental pragmatics

## Abstract

While some aspects of pragmatic competence are known to decline with age, for metaphor skills the evidence is inconclusive, possibly due to heterogeneity in the assessment tools. Furthermore, the previous literature on age‐related changes in pragmatic skills has rarely considered the role of Theory of Mind (ToM), which is described as one of the main factors affecting metaphor across theoretical and experimental studies in children and clinical populations. This study aimed at elucidating age‐related differences in metaphor understanding and the interplay between metaphor skills and ToM in middle‐aged and older adults with a fine‐grained approach. Participants (*n* = 201, age range 54–93) completed tasks assessing ToM and metaphor understanding. On the one side, we used the Physical and Mental Metaphors task, to distinguish between different types of metaphor (*physical*, such as “Lifeguards are lizards,” meaning that they lie in the sun, vs. *mental*, such as “Adolescents are pendulums,” meaning that they are emotionally unstable) and different aspects of metaphor understanding, namely, *accuracy* in finding a link between topic and vehicle versus type of *interpretation* (from physical to psychological). On the other side, we analyzed two aspects of ToM skills: the *accuracy* in mental state understanding and the *intentionality*, defined as the degree of mental state attribution, assessed with the Strange Stories and the Animated Social Triangles task, respectively. Structural equation models indicated a decline in metaphor skills with advancing age. Furthermore, we found that ToM is involved in metaphor understanding in a specific fashion. While higher ToM accuracy explained better metaphor accuracy, higher ToM intentionality explained better performance in the interpretation of mental, but not physical, metaphors. These findings suggest that age‐related differences in pragmatics extend to metaphor skills and that ToM plays a role in metaphor comprehension in older age, with a division of labor where the ability to understand what others think is key to spotting a metaphorical link, but the greater tendency to attribute mental states is what specifically helps when it comes to grasping the psychological nuances of a metaphor.

## Abstract 2

This study shows age‐related differences in metaphor understanding in a cohort of middle‐aged and older adults. We also show that Theory of Mind is involved in metaphor understanding in a specific fashion in older age. While mental state understanding is key to grasping the link between the elements of a metaphor, the degree of mental state attribution is key to interpreting the psychological nuances of metaphorical meaning.

## 1 Introduction

Pragmatic abilities include a large set of skills that allow people to communicate effectively by going beyond the literal meaning of words and adjusting to the interlocutor's needs in conversation (Wilson & Sperber, 2004). Pragmatics is key for managing successful daily interactions and creating (and maintaining) social relationships (Agostoni et al., 2021; Cummings, 2016; Snow & Douglas, 2017) and is clearly pivotal for older people, who are at higher risk of social isolation and loneliness. There is growing evidence that older adults starting as early as 50−60 years show reduced pragmatic skills, both in production and comprehension (Bambini et al., 2021; Hilviu, Gabbatore, Parola, & Bosco, 2022; Messer, 2015). However, for some receptive pragmatic aspects such as metaphor understanding the literature still debates over possible age‐related effects and the existence of progressive difficulties in older people. In this paper, we focus precisely on metaphor skills, examining how this ability is affected by old age and exploring the role of Theory of Mind (ToM).

In the pragmatic framework, metaphors are nonliteral uses of language displaying a major gap between the literal and the intended meaning. They involve a conceptual shift (Carston, 2010), captured by the traditional definition as the description of one element in terms of another. For instance, in “Adolescents are pendulums” the concept evoked by the vehicle word “pendulums” does not refer to objects whose movements are oscillatory, but is broadened to refer primarily to people's changing mental states (Wilson & Carston, 2007). To date, limited research examined how metaphor comprehension skills change in late adulthood and healthy aging, obtaining contrasting results (Bartczak, 2017). While some studies indicated a decline in metaphor skills (Daniluk & Borkowska, 2020; Morrone, Declercq, Novella, & Besche, 2010; Sundaray, Marinis, & Bose, 2018), other studies highlighted preserved or even enhanced metaphorical reasoning in older people (Newsome & Glucksberg, 2002; Qualls & Harris, 2003). The inconclusiveness in findings is likely due to the differences in the components of the metaphorical comprehension process examined across studies. Some studies focused on the appreciation of the metaphorical meaning of sentences by investigating priming effects (Morrone et al., 2010), while others requested participants to judge whether sentences/word pairs were literally true or false (Kavé, Gavrieli, & Mashal, 2014), or how much they were plausible/familiar (Mashal, Gavrieli, & Kavé, 2011). Most studies focused on multiple‐choice tasks, asking participants to select the correct meaning of a metaphor among different options (e.g., Daniluk & Borkowska, 2020), while only a few adopted verbal explanation tasks, where participants are asked to explain or paraphrase a metaphor. This latter response format arguably requires additional expressive language skills; however, it is largely considered a comprehension task, with the additional advantage of being more sensitive than other tasks in detecting impairment in metaphor comprehension and allowing to establish with more confidence whether the metaphors were understood or not (Kalandadze, Bambini, & Næss, 2019). Interestingly, Gregory and Waggoner (1996) compared performances in multiple‐choice versus verbal explanation tasks in younger and older adults. Their findings indicated that age‐related differences emerged exclusively in the verbal explanation task but not in the forced‐choice task, in which both age groups performed very highly, with over 90% accuracy (see also Bambini et al., 2021).

Furthermore, heterogeneity in results might depend on which aspect of metaphor comprehension is at stake. For instance, some studies investigated how young and older adults interpret metaphors (Mashal & Coblentz, 2014). The authors examined the typology of explanations for metaphorical word pairs and concluded that older people were more creative and integrative than young adults in their explanations. These results echoed those found by other researchers focusing on age‐related differences in style (e.g., literal/analytic vs. poetic/synthetic), rather than correctness, in interpreting metaphors (Kramer & Woodruff, 1984; Szuchman & Erber, 1990). Overall, these findings suggested that, besides the accuracy of the inferred meaning, also how metaphors are interpreted may change with aging, supporting a multicomponential view of metaphor skills.

Another relevant issue to consider is the role of other cognitive skills in metaphor understanding. There is evidence indicating that older adults’ possible difficulties in metaphor skills might be associated with the well‐known decline in cognitive functioning, especially inhibitory control and working memory (Bambini et al., 2021; Bartczak, 2017). Very little attention, however, has been paid to the role of ToM in how older adults understand metaphors. Conversely, the role of ToM in pragmatics is at the center of a growing interest in research in other populations (Cummings, 2013; Norbury, 2005; Rossetti, Brambilla, & Papagno, 2018). ToM is a multifaceted construct globally referring to the ability to attribute mental states, such as thoughts, emotions, and desires, to oneself and others, to anticipate, explain, and influence others’ behaviors (Apperly, 2012). In childhood, it is often assessed using the false‐belief paradigm, reflecting the ability to separate one's knowledge from the other's knowledge, appreciating that people may have wrong beliefs and behave accordingly (Wellman, Cross, & Watson, 2001). This is a pass‐fail test, which is generally not suitable beyond preadolescence, as individuals perform at ceiling. However, ToM continues to develop and change across the lifespan, and advanced ToM tasks have been created for the assessment of individual differences beyond school age (Breil & Böckler, 2020; Miller, 2012). In adulthood and aging advanced ToM skills are assumed to include a variety of aspects, such as making inferences from nonverbal cues, interpreting ambiguous social situations, recognizing social blunders, and ascribing mental states to nonhuman objects (Osterhaus & Bosacki, 2022).

Considering that the ability to detect the speaker's communicative intention is a core aspect of pragmatic competence, several authors argued that ToM underlies pragmatics (Hyter, 2017; Sperber & Wilson, 2002). To date, a link between pragmatics and ToM is generally acknowledged, even if the exact nature of the relationship between the two constructs is still under debate (Bosco, Tirassa, & Gabbatore, 2018; Fairchild & Papafragou, 2021). Focusing on metaphor, there is growing evidence of a link with ToM in childhood and clinical populations. For instance, a series of studies identified both cross‐sectional and longitudinal relations between ToM and metaphor understanding in middle childhood (Del Sette, Bambini, Bischetti, & Lecce, 2020; Lecce, Ronchi, Del Sette, Bischetti, & Bambini, 2019), with stronger associations in younger than older children (Bambini et al., 2024; Lecce et al., 2019; Tonini et al., 2023). While there is evidence that ToM may serve as a springboard for achieving metaphorical competence in children at certain ages, there is also evidence from training studies that metaphor skills might drive advanced ToM (Del Sette, Bambini, Tonini, & Lecce, 2024). On a different note, studies in patients with schizophrenia and traumatic brain injuries suggested that difficulties in metaphor understanding could be related to impairments in ToM (Champagne‐Lavau & Joanette, 2009; Gavilán & García‐Albea, 2011). Overall, researchers argue that ToM abilities play a role in extracting the communicative intentions behind the literal meaning of words when nonliteral language is used. When ToM abilities are still developing (in children) or are impaired (in clinical populations), difficulties in metaphor understanding are likely to occur. Additionally, recent evidence showed that the relationship between ToM and metaphor comprehension depends on the type of metaphor and the specific ToM task adopted. ToM is more involved in metaphors that express mental characteristics compared to those expressing physical characteristics (Canal et al., 2022; Lecce et al., 2019), and its role might emerge with some ToM tasks (especially verbal ones) but not with others (Canal et al., 2022).

Crucially, ToM skills are known to decline with aging (Fernandes, Barbosa, Martins, & Marques‐Teixeira, 2021; Henry, Phillips, Ruffman, & Bailey, 2013). Older people are less able to understand others’ mental states and to use this awareness to comprehend complex social situations (Cavallini, Lecce, Bottiroli, Palladino, & Pagnin, 2013; Fischer, O'Rourke, & Thornton, 2017). On the one side, some studies suggested that language and executive functions (EF) are positively related to older adults’ ToM skills. For instance, Charlton and colleagues (2009) found that both verbal intelligence (vocabulary and similarities tasks) and fluid intelligence (executive functions and processing speed) mediated the link between age and ToM in adults aged from 50 to 90 years old. Furthermore, Slessor, Phillips, and Bull (2007) found that the initial similarity in ToM performance between younger and older participants disappeared when statistically controlling for vocabulary knowledge. The authors concluded that older adults’ advantage in vocabulary could compensate for age decline in ToM. On the other side, the decline in ToM has been recognized as partly independent of older people's functioning in other cognitive domains (Akarsu, 2018; Kong, Currie, Du, & Ruffman, 2022). In turn, the impairments in ToM affect older people's social functioning, in terms of reduced quality and variety of the social network (Krendl, Kennedy, Hugenberg, & Perry, 2022; Lecce et al., 2017), and lower satisfaction with social exchanges (Blanke, Rauers, & Riediger, 2016). Notably, as the pattern of decline in ToM varies across ToM tasks (Bottiroli, Cavallini, Ceccato, Vecchi, & Lecce, 2016; Klindt, Devaine, & Daunizeau, 2017; Slessor et al., 2007), it is important to consider different facets of this skill (Breil & Böckler, 2020).

### 1.1 The present study

From the literature review presented above, it emerges that there is still confusion on how (and if) metaphor skills change in adulthood and aging. Although it is acknowledged that metaphor understanding is multifaceted, studies examined a single dimension at once. Furthermore, the impact of sociocognitive skills on metaphor understanding is poorly explored in healthy aging. Therefore, our first aim was to test the hypothesis of an age‐related decline in metaphor skills, adding fresh evidence to the debate on this issue. To capture the decline moving from middle to late adulthood, we recruited participants from 50 to over 90 years old. The second aim of the study was to investigate the associations between metaphor and ToM skills in middle and older adults adopting a fine‐grained approach that took into account previous evidence indicating that these two skills should not be considered as monolithic.

To pursue these aims, and given that different metaphor aspects might change differently over aging, we adopted the Physical and Mental Metaphor (PMM) task (Lecce et al., 2019), which allows to distinguish between (i) two types of metaphorical expressions, *mental* and *physical* metaphors; and (ii) two aspects of metaphorical processing, *accuracy* and *interpretation*. For (i), the PMM includes nominal metaphors (X is Y) with minimal context in which persons (X, the topic) are associated with nonhuman entities (Y, the vehicle) based either on physical features (e.g., “Lifeguards are lizards,” meaning that they lie in the sun) or psychological features (e.g., “Adolescents are pendulums,” meaning that they are emotionally unstable). Hence, inferences on appearance features or behaviors of the topic are needed to understand physical metaphors, whereas inferences on psychological characteristics of the topic are needed in mental metaphors. For (ii), the PMM assesses separately the capacity to understand the link between the topic and the vehicle of the metaphor (*metaphor accuracy*) and the type of interpretation provided when explaining the meaning of metaphors, on a scale from the physical to the psychological dimension (*metaphor interpretation*). These distinctions allowed us to take into account cases in which people give a plausible explanation of a metaphor but fail to grasp its psychological (vs. physical) implications (e.g., by explaining the metaphor “Adolescents are pendulums” as “They move back and forth”).

A fine‐grained analysis was also adopted for ToM, based on Osterhaus and Bosacki's theoretical framework, which emphasizes that advanced ToM “is a multifaceted and nuanced construct” (2022, p. 20). We focused and analyzed separately two aspects of ToM: the *accuracy* in mental state understanding, that is, the ability to correctly explain social behavior in complex scenarios; and the *intentionality* attribution, as the degree to which participants ascribe complex intentional mental states and use elaborated psychological language. The latter aspect reflects the spontaneous tendency to attribute mental states, and some studies suggested that this tendency, more than the raw accuracy in deliberate mental reasoning, can explain success or failure in social situations (Klin, 2000; Lecce, Ceccato, & Cavallini, 2019; Warnell & Redcay, 2019). Although correlated, these two aspects of ToM are distinct: accuracy in inferring other people's mental states differs from the ascription of mental states to others, with the latter being independent of the former (Basile, Lecce, & van Vugt, 2022; Van den Stock et al., 2019). Indeed, it is possible to have a high degree of attribution of mental states while inferring a wrong mental state, or to refer rarely to mental states while being able to infer the correct mental state in a given situation (Rice & Redcay, 2015). For instance, on the one hand, a person can believe that their partner is keeping the door open for an attractive stranger because the partner is trying to impress the stranger and obtain a chance to exchange glances or chat, while in fact the partner is simply being polite (high attribution—low accuracy). On the other hand, when seeing a college student struggling to keep their eyes open during a morning lecture, one can easily infer that the student is tired, without perhaps realizing that they are working hard because they want to pass the exam as they believe that the exam is really important for the future career that they want to pursue (low attribution—high accuracy). To assess these two aspects of ToM, in this study we adopted the Strange Stories task (White, Hill, Happé, & Frith, 2009) for accuracy and the Animated Social Triangles (AST) task (Castelli, 2002) for intentionality attribution (see the Method section for details on these instruments).

Based on the literature that documented the fragility of pragmatic skills in aging (Bambini et al., 2021; Hilviu et al., 2022), we expected that age would impact metaphor understanding. Furthermore, following the idea put forward in Relevance Theory that understanding metaphors requires inferring how another person sees the world (Happé, 1993), we expected that the link between metaphor and ToM would surface in aging as well. Specifically, capitalizing on the evidence that different pragmatic tasks have a different burden on ToM (Bischetti, Ceccato, Lecce, Cavallini, & Bambini, 2023; Lecce et al., 2019; Tonini et al., 2023), we hypothesized a greater involvement of ToM skills in mental rather than physical metaphors. Although both types of metaphor are to some extent expected to be related to ToM when considered in real usages (Lecce et al., 2019), here we focused on the further mindreading processes that might be related to the specific content of the message and that might be greater when this refers to internal states of the topic as in mental metaphors. Also, we reasoned that different ToM aspects (i.e., accuracy vs. intentionality) may distinctively affect different facets of metaphor comprehension. Due to the lack of previous evidence, we did not have any strong expectations. Still, we deemed that ToM accuracy should be crucial for metaphor accuracy, in line with previous studies that focused on these two facets (Bambini et al., 2021; Del Sette et al., 2020; Gavilán & García‐Albea, 2011). For ToM intentionality, we speculated that this facet could be more relevant for metaphor interpretation than for accuracy, as the spontaneous attribution of mental states may translate into a greater psychologically oriented interpretation of metaphors.

To examine the links between the different constructs, we used a structural equation modeling (SEM) approach, which allows to test the fit of hypothesized theoretical models to observed data. SEM aims at testing the causal assumptions derived from prior studies and scientific knowledge using correlational data, to examine how well the observed data are consistent with the model‐implied associations. Using SEM to assess the fit of a specified model “does not ‘prove’ the causal assumptions, but it makes them tentatively more plausible” (Bollen & Pearl, 2013, p. 309). Moreover, SEM allows testing complex models including a number of variables simultaneously, highlighting unique relationships while controlling for the effect of the other variables in the model. Furthermore, compared to linear regression, SEM also allows to (1) estimate error variances; (2) have a model overview, with fit indices and indirect effects taken into account, rather than a list of associations between a range of predictors and a single dependent variable (Iacobucci, 2009; Kline, 2011). SEM models include a measurement model, which relates the observed indicators to the latent variable (i.e., a confirmatory factor analysis ‐ CFA ‐ linking the items to the theoretical construct), and a structural path model, which relates the constructs (either latent variables or observed variables) to each other. Scholars recommend assessing the fit of the measurement models first, and then moving to examine the structural model by fitting it against the data. If one or more measurement models do not satisfactorily fit the data, they should be respecified before moving to assess the structural model (Brown, 2015; Iacobucci, 2009; Kline, 2011). Here, we followed this recommended procedure and assessed first the measurement models and then the structural model. Moreover, thanks to the SEM approach, the relationship between different metaphor aspects and different ToM aspects was investigated while controlling for educational attainment and vocabulary knowledge, two variables that were previously associated with better performance in both metaphor and ToM (Champagne‐Lavau, Monetta, & Moreau, 2012; Li et al., 2013; Slessor et al., 2007; Szepietowska & Filipiak, 2021).

## 2 Method

### 2.1 Participants

We recruited 201 adults aged from 54 to 93 years old (*M* = 70.37, *SD* = 7.31; Female: 74%). Female and male participants did not differ in age, *M* = 70.14, *SD* = 7.65 and *M* = 71.02, *SD* = 6.29, respectively, *t*(199) = 0.75, *p* = .455. The age range was selected based on past studies suggesting that both metaphor skills and ToM start to decline approximately from the age of 50 (Gourlay, Collin, D'Auteuil, Jacques, & Scherzer, 2022; Kong et al., 2022; Monetta, Ouellet‐Plamondon, & Joanette, 2007). Educational attainment was measured on a 6‐point scale (0 = no degree; 1  =  elementary school; 2 = middle school; 3 = high school; 4 = bachelor's degree; 5  =  master's degree; 6 = Ph.D. and/or post‐graduate degree). All participants were native speakers of Italian, independently living, with no history of neurological or psychiatric illness, and were not taking any medications that could affect cognitive functioning. Participants were prescreened with the Mini‐Mental State Examination (Folstein, Folstein, & McHugh, 1975; Magni, Binetti, Bianchetti, Rozzini, & Trabucchi, 1996); they all scored above the 24‐point cut‐off. Participants were unpaid volunteers recruited through sociocultural senior centers, personal contacts, word of mouth, and local advertisements.

Tests were administered in a quiet room, either at recreational centers or at the participant's own home, under the supervision of a research assistant preliminary trained on the administration procedure. The order of the tasks was randomized. The study was approved by the Ethics Committee of the Department of Brain and Behavioral Sciences of the University of Pavia. Written informed consent was obtained from all participants before testing.

### 2.2 Measures

#### 2.2.1 Metaphor skills

We used the PMM task, which has been successfully employed in adulthood and aging (Bambini et al., 2022; Bambini et al., 2020; Canal et al., 2022). This task measures the ability to understand and interpret nominal metaphors presented in the predicative form in a minimal context via verbal explanation. The task includes 14 metaphors belonging to two different types: physical metaphors (*N* = 7) and mental metaphors (*N* = 7). The first type requires inferences about physical or behavioral aspects of the metaphor's topic, whereas the second type requires inferences about psychological aspects. All the metaphors were presented in the written format and participants were asked to write down the responses in a booklet (Fig. 1, left; see also Supplementary Materials, Section A). Following the original scoring criteria (Del Sette et al., 2020), answers were coded on two dimensions: *accuracy* and *interpretation*. Accuracy reflects the ability to articulate the meaning of the metaphorical expression, based on its salience, that is, to identify the salient meaning of the metaphor on the basis of conventionality, frequency/familiarity, and prototypicality (Del Sette et al., 2020). Scores were as follows: 0 points for incorrect, don't know, and literal responses; 1 point for partially correct responses and responses focusing on nonsalient features of the vehicle; and 2 points for fully correct and salient responses. Average accuracy scores were computed for all metaphors (Tot accuracy), and for physical and mental metaphors separately, ranging from 0 to 2. To validate the scoring procedure, a salience analysis was performed, which clustered in features the responses generated by an independent sample of young adults presented with the PMM metaphors. The salience analysis confirmed that the answers coded 2 points based on the scoring manual were those containing the most common features generated by the participants (see the Supplementary Material, Section A; and Del Sette et al., 2020 for a similar approach). Interpretation reflects the type of interpretation (on a scale from physical to psychological) and is based on the use of mental state terms, that is, words reflecting emotions, thoughts, and desires, in explaining the metaphor.^1^ Responses were coded as follows: 0 points for answering “don't know”; 1 point for answers referring to physical attributes; 2 points for answers presenting ambiguous attributes, not identifiable as either mental or physical; and 3 points for answers referring to psychological attributes. Average interpretation scores were computed for all metaphors (Tot interpretation) and for physical and mental metaphors separately, ranging from 0 to 3. See Supplementary Material, Section A for scoring examples.

**Fig. 1:**
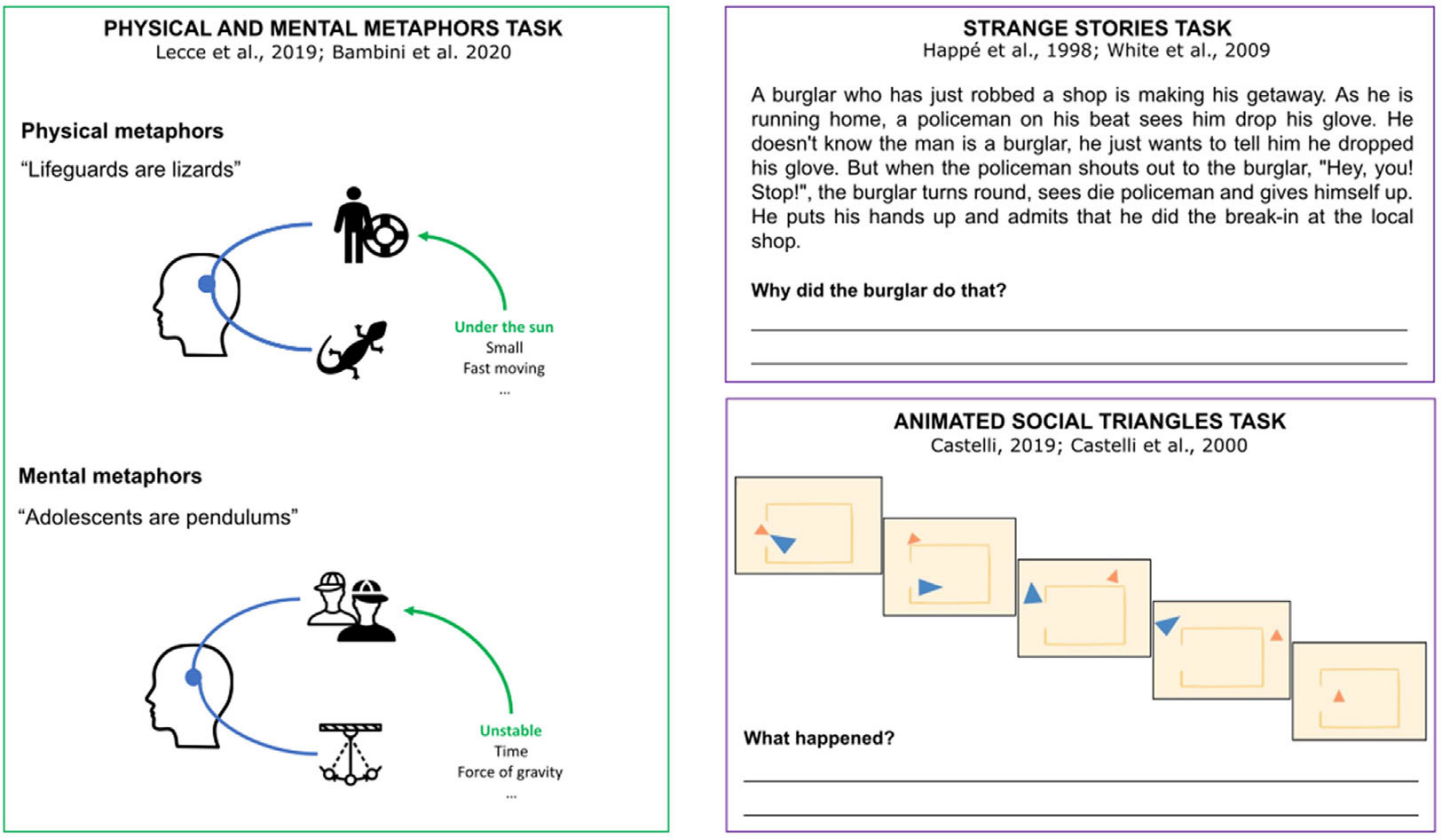
Visual description of the tasks. *Left*: Physical and Mental Metaphors task. The figure depicts the inferential processes supporting the understanding of physical and mental metaphors—based on an example for each type. The task was administered in the written format, with no visual aid. *Right*: ToM tasks. On the top, one of the five stories assessing ToM accuracy (Strange Stories). On the bottom, a sequence of screenshots from one of the three video clips assessing ToM intentionality (Animated Social Triangles).

### 2.3 Theory of Mind

It was assessed using the Strange Stories (SS) task and the Animated Social Triangles (AST) task (Fig. 1, right). The SS task is an advanced ToM test (Happé, Winner, & Brownell, 1998; White et al., 2009) commonly used in research on aging (Osterhaus & Bosacki, 2022). It evaluates the ability to understand the mental states underlying people's social behaviors in complex social scenarios. We selected five mental state stories with different scenarios: double bluff (*N* = 2), persuasion (*N* = 2), and misunderstanding (*N* = 1). According to the scoring criteria, answers that explain characters’ actions considering their mental states were scored 2, partially correct answers were scored 1, and 0 was given to incorrect answers. The total score was transformed into a percentage of accuracy. A second rater coded 30% of the protocols and interrater reliability was excellent (Cohen's *k* = .91). Disagreements between the raters were then discussed until a consensus was reached. The AST (Castelli, Happé, Frith, & Frith, 2000) is a video‐based ToM task that evaluates the extent to which people attribute intentionality to geometric shapes (triangles) based on their movements. This task allows to assess people's spontaneous ascription of mental states, independently of whether they do so accurately or not (Eddy, 2019). We selected three videos in which the triangles behaved in such a way as to imply teasing and deceiving (i.e., ToM/cognitive animations; Castelli, 2002; Ceccato, Lecce, Cavallini, Vugt, & Ruffman, 2019). After watching each video clip, participants were asked to write down what happened in the clip. Answers were then coded for intentionality, reflecting the degree to which participants described the triangles’ movements in terms of complex intentional mental states. Following the original scoring procedure, the intentionality score ranged from 0 (no intentionality attributed, movements without purpose) to 5 (one triangle manipulating the mental state of the other triangle). The three items were collapsed into a percentage score. A second rater coded on 30% of the protocols and interrater reliability was satisfactory (Cohen's *k* = .73). Disagreements between the raters were then discussed until a consensus was reached.

### 2.4 Vocabulary

We used the verbal meaning subscale from the Primary Mental Abilities battery (Thurstone & Thurstone, 1963). This test requires participants to identify the correct synonym of 50 target words by choosing from four given alternatives within 8 min. The test was administered in a paper‐and‐pencil format. The total score ranged from 0 to 50.

### 2.5 Data analysis

First, we performed correlation analyses to inspect zero‐order relationships between the investigated variables. Second, we examined the measurement models (i.e., CFAs, see Brown, 2015 and Kline, 2011) of the PMM, SS, and AST tasks. Finally, we used SEM to examine the unique contribution of ToM skills in metaphor understanding, taking into account age, education, and vocabulary as control variables. Based on the literature, we specified a structural model in which we regressed PMM accuracy and PMM interpretation onto age, education, vocabulary, and ToM tasks. PMM accuracy and interpretation were specified to covary. ToM was regressed onto age, education, and vocabulary. Vocabulary was regressed onto age and education. PMM accuracy score and ToM tasks were modeled as latent factors based on results from the measurement model analyses, while PMM interpretation scores, age, education, and vocabulary were modeled as observed variables.

The weighted least square mean and variance adjusted (WLSMV) estimator was used as some of the tasks had categorical items. Model fit was assessed using common indices and suggested cut‐off (see Supplementary Material, Table S4). Analyses were conducted using SPSS 19 (IBM Corp., 2010) for descriptive and correlational analyses, and Mplus Version 7 (Muthén & Muthén, 2012) for measurement models and SEM.

### 2.6 Data availability

The dataset, together with the syntax codes used in MPlus for the analyses, is available in the Open Science Framework: https://osf.io/3dtna/?view_only=4e65b337e41840ac890e4f2179cb3a44.

## 3 Results

Descriptive statistics and bivariate correlation analysis are presented in Table 1. Inspecting zero‐order relationships, we found the expected pattern of age‐related decline. Namely, both metaphor and ToM skills were negatively related to age. Also, both education and vocabulary were positively related to metaphor and ToM skills, indicating that people with higher educational attainment and linguistic ability have better performances in both metaphor processing and ToM tasks.

**Table 1:** Descriptive statistics and zero‐order correlation

	Descriptive				Correlation									
	*M*	*SD*	*Possible range*	*Actual range*	*Edu*	*Voc*	*SS*	*AST*	*PMM acc*			*PMM int*		
									*Overall*	*Mental*	*Physical*	*Overall*	*Mental*	*Physical*
***Age***	70.37	7.31	−	54−93	−.33^***^	−.42^***^	−.32^***^	−.19^**^	−.43^***^	−.31^***^	−.48^***^	−.33^***^	−.40^***^	−.09
***Education***	2.92	1.21	0–6	0–6	−	.53^***^	.35^***^	.14	.37^***^	.35^***^	.32^***^	.29^***^	.37^***^	.05
***Vocabulary***	42.37	8.99	0–50	6–50		−	.61^***^	.17^*^	.54^***^	.47^***^	.51^***^	.37^***^	.47^***^	.06
***SS***	63.55	21.96	0–100	0–100			−	.21^**^	.52^***^	.44^***^	.49^***^	.30^***^	.40^***^	.03
***AST***	58.86	15.94	0–100	0–100				−	.29^***^	.31^***^	.22^**^	.26^***^	.28^***^	.12
***PMM acc***														
*Overall*	1.32	0.40	0−2	0−1.9					−	.93^***^	.89^***^	.52^***^	.70^***^	.05
*Mental*	1.26	0.48	0−2	0−2						−	.62^***^	.56^***^	.72^***^	.09
*Physical*	1.38	0.42	0−2	0−2							−	.37^***^	.54^***^	.00
***PMM int***														
*Overall*	1.81	0.33	0−3	0.9−2.7								−	.86^***^	.73^***^
*Mental*	2.42	0.48	0−3	0.9−3									−	.36^***^
*Physical*	1.19	0.31	0−3	0.4−2.4										−

*Note*. *N* = 201. Data refer to the PMM with 14 items (seven mental metaphors, seven physical metaphors).

Abbreviations: AST, Animated Social Triangles task; Edu, education; PMM acc, PMM task, accuracy score; PMM int,  PMM task, interpretation score; SS, Strange Stories task; Voc, vocabulary.

^*^ *p* < .05; ^**^ *p* < .01; ^***^ *p* < .001.

The measurement models are presented in detail in Supplementary Material, Section B. Summing up, for PMM accuracy, the final latent structure was composed of a single dimension made up of both physical and mental metaphors (13 items), χ^2^(65) = 107.16, *p* < .001, χ^2^/df ratio = 1.65, CFI = 0.95, TLI = 0.94, RMSEA = .06, 90% CI [.04, .08]. Considering the large number of items, we followed Little's recommendations and decided to use parcels instead of single items. Models based on parceled data are more parsimonious and reduce sampling error compared to item‐level data (Koran, 2020; Little, Cunningham, Shahar, & Widaman, 2002). Hence, for PMM accuracy, we created three parcels with a balancing approach (i.e., grouping the items based on their factor loadings; see Table S4) (Little, Rhemtulla, Gibson, & Schoemann, 2013). For the interpretation score, no satisfying latent structure emerged, neither bidimensional nor unidimensional. Therefore, we used the observed variables (i.e., the average scores described in the Method section and presented in Table 1), keeping mental and physical metaphors separate, as previously done in the literature (Del Sette et al., 2020; Lecce et al., 2019). Therefore, we entered in the SEM two interpretation scores, one for physical metaphors (6 items) and one for mental metaphors (7 items), as observed variables, using the average scores.

Both ToM tasks revealed satisfying unidimensional latent structures: for SS, χ^2^(5) = 2.38, *p* = .795, χ^2^/df ratio = 0.48, CFI = 1.00, TLI = 1.05, RMSEA = .00, 90% CI [.00, .06]. For AST, the model was just‐identified, hence fit indices did not apply (Brown, 2015). However, the factor loadings were all significant, *p*s < .001.

Finally, SEM analysis indicated excellent fit indices, χ^2 ^(81)  =  78.71, *p* = .551, χ^2^/df ratio = 0.97, CFI  = 1.00, TLI = 1.00, RMSEA  = .00, 90% CI [.00, .04]. Age and education were negatively associated, *p < *.001, as older people generally had lower levels of educational attainment. Also, higher vocabulary was predicted by lower age, *p* < .001, and higher education, *p* < .001. Results also showed that SS, but not AST, was significantly predicted by vocabulary, *p *< .001. Age and education had no direct effects on any of the ToM tasks, *p*s ≥ .081 and *p*s ≥ .393, respectively. Focusing on the aims of the study, data showed that age negatively predicted PMM accuracy, *p * =  .024, and interpretation of mental metaphor, *p*  =  .007, but not interpretation of physical metaphors, *p* = .638. Education and vocabulary had no effects on metaphor skills, neither accuracy nor interpretation, *p*s ≥ .085. With respect to the role of ToM on metaphor skills, PMM accuracy was predicted by the SS, *p * =  .011, but not by the AST, *p * =  .080. Interpretation of mental metaphors was significantly predicted by AST, *p * =  .047, but not SS, *p * =  .303, while interpretation of physical metaphors was not significantly explained by any of the two ToM tasks, *p*s ≥ .200. Globally, the model accounted for 56% of the variance in PMM accuracy, 35% in the interpretation of mental metaphors, and 3% in the interpretation of physical metaphors (Fig. 2).

**Fig. 2:**
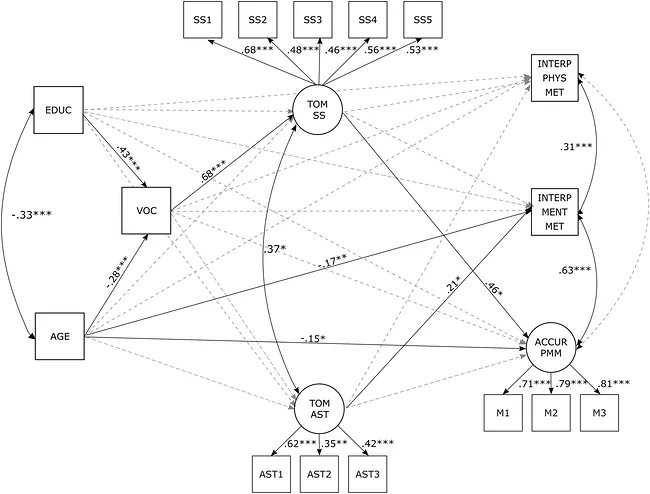
SEM model depicting the relationships between age, education, vocabulary, ToM, and metaphor skills. EDUC, education; INTERP MENT MET, interpretation score of mental metaphors in the PMM task; INTERP PHYS MET, interpretation score of physical metaphors in the PMM task; PMM ACCUR, accuracy latent factor of the PMM task; TOM AST, Animated Social Triangles task; TOM SS, Strange Stories task; VOC, vocabulary. Standardized coefficients are reported. Black lines represent paths with *p* < .05. Dashed gray lines represent paths with *p* > .05. *p < .05; **p < .01; ***p < .001.

## 4 Discussion

The current study had two main aims: first, to provide fresh evidence on the decline in metaphor skills across middle and late adulthood; and second, to examine the role of ToM in metaphor skills in this phase of life. Two aspects of the study were particularly innovative: first, the focus on the sociocognitive correlates of metaphor skills, and second, the adoption of a fine‐grained approach in examining both metaphor and ToM constructs. On the one side, based on studies suggesting that different aspects of metaphor processing may be differently impacted by age, we took into account metaphor type (mental vs. physical) and aspect of processing (accuracy vs. interpretation). On the other side, based on research highlighting the need to consider ToM as a multifaceted construct, we differentiated between accuracy in understanding complex social situations and the degree of attribution of mental state. We expected to find an age‐related decline in metaphor skills and an association between metaphor understanding and ToM. Concerning the latter aspect, we anticipated (i) a specific involvement of ToM depending on the type of metaphor, and (ii) a specific role of different ToM aspects on separate facets of metaphorical understanding. The findings generally confirmed our hypotheses. We found that metaphor skills decline in aging, and results pointed to the specificity of the relationship between metaphor and ToM in this phase of life. More precisely, we identified a division of labor between the two ToM aspects—mental state accuracy and degree of mental state attribution—in supporting metaphor understanding, with the former being associated with metaphor accuracy and the latter with metaphor interpretation of mental, but not physical, metaphors. Below is a detailed explanation of each result.

Regarding the debate on the effects of aging on metaphor skills, we found that with advancing age metaphor skills—measured as accuracy in understanding and explaining the meaning of metaphors—decline. This finding aligns with recent studies evidencing a progressive decline in global pragmatic skills in typical aging (Bambini et al., 2021; Bischetti et al., 2024; Hilviu et al., 2022) and fits well with some preliminary evidence indicating that the difficulties in metaphorical processing may be due to age‐related differences in neural activation (Mejía‐Constaín et al., 2010). To date, empirical evidence on this topic has been sparse and conflicting (e.g., Daniluk & Borkowska, 2020). Most of the research investigating metaphor skills in younger, middle, and older adults adopted multiple‐choice tasks, but this test format may conceal subtle individual differences. For example, in a study on the Italian population, performances in a multiple‐choice metaphor task were extremely high, with people from 20 to 79 years old obtaining accuracy scores over 90% (Zanini, Bryan, De Luca, & Bava, 2005), potentially hiding age‐related differences. The present study extends the available literature by using a verbal explanation task, rarely used in aging but arguably a more sensitive measure (Bambini et al., 2020). Verbal explanation tasks require paraphrasing metaphorical sentences, hence they offer a greater insight into people's metaphorical understanding, even if they conceivably load more on verbal and executive skills (Kalandadze et al., 2019). Furthermore, the choice of a verbal explanation task offers a greater ecological value compared to multiple‐choice tasks, as in daily life people have to understand, and potentially explain, figures of speech they read or hear, rather than selecting the right alternative.

The second aim of the study was to examine the role of ToM in metaphor skills by separating accuracy and intentionality aspects. Several studies highlighted the importance of assuming a multicomponential view of ToM and measuring more than a single facet (Osterhaus & Bosacki, 2022), as different aspects of ToM may fulfill different functions and show specific age‐related patterns (Klindt et al., 2017). For instance, in a recent study, Basile and colleagues found that movement synchronization between participants enhanced mental state attribution in the AST task but had no effect on the accurate identification of the partner's emotions and intentions (Basile et al., 2022). In research on aging, enhanced motivation was able to improve ToM performance in a task assessing the accurate identification of mental states in social scenarios similar to the Strange Stories (i.e., Faux Pas task), but not in the AST task (Zhang et al., 2018). Our findings indicated that ToM accuracy, but not intentionality, plays a crucial role in metaphor comprehension, over and above the direct effects of age, education, and language skills. On the contrary, intentionality attribution explained, over and above the direct effect of the other variables, the interpretation of mental, but not physical, metaphors. These results suggest a differential involvement of ToM skills in metaphor processing in middle‐aged and older adults: while the ability to understand what others think helps in identifying a metaphorical link, the greater tendency to attribute mental states is what helps grasp specifically the psychological nuances of a metaphor. Taken together, the present findings favored the claim of a different burden on ToM skills, depending on the features of the pragmatic task, in line with previous studies in developmental and aging research (Bischetti et al., 2023; Domaneschi & Bambini, 2020; Lecce et al., 2019; Tonini et al., 2023).

Nevertheless, a further consideration is that our data did not highlight a clear effect of age on ToM skills in this sample: while correlational analysis showed that age was negatively associated with both ToM tasks, only mediated effects through vocabulary skills on Strange Stories emerged in the SEM analysis, possibly reflecting the importance of language skills as a cognitive underpinning of ToM in aging (Slessor et al., 2007). Related to language skills, unexpectedly, we found that age was negatively related to vocabulary. Despite the cognitive decline with age, vocabulary knowledge is usually maintained or even enhanced in older adults (Kavé, 2022; Verhaeghen, 2003). The advantage of older compared to younger adults, however, has been widely documented when comparing extreme age groups (e.g., Ben‐David, Erel, Goy, & Schneider, 2015), but it is less evident when analyzing only older people samples, as we did (Bowles & Salthouse, 2008). Furthermore, as our vocabulary task included a time limit (8 min), we speculate that this may have impaired the performance of the oldest participants, who may need more time to complete the task.

Parallel to the importance of considering different ToM aspects, our study also highlights the importance of considering different metaphor aspects. Here, we focused on accuracy and type of interpretation in understanding metaphors, which were already reported to differ in developmental trajectories (Del Sette et al., 2020; Lecce et al., 2019). We also examined the role of metaphor type by distinguishing between mental and physical metaphors, which also were already shown to differ in the acquisition of metaphor skills (Del Sette et al., 2020; Gentner, 1988; Tonini et al., 2023; Winner, Rosenstiel, & Gardner, 1976). These distinctions between score aspects and metaphor types were crucial also for aging when examining the sociocognitive predictors of metaphor interpretation skills. Interestingly, while mental metaphor interpretation was affected by age and was related to ToM intentionality, none of the predictors considered here affected the interpretation of physical metaphors, which might represent a relatively independent skill, possibly linked to other abilities, such as, for instance, mental imagery. Indeed, there is a large literature claiming that imagery skills can support metaphor understanding (Bambini et al., 2024; Carston, 2018; Ifantidou, 2021). Since physical metaphors refer to the appearance or behavioral aspects of the topic, their comprehension might rely on visual images to a larger degree compared to metaphors referring to internal states, as suggested by recent neurophysiological evidence obtained by comparing the two metaphor types (Canal et al., 2022). Conversely, data suggested a one‐factor solution for accuracy. In other words, the ability to correctly explain the meaning of a metaphor seems relatively independent from its physical versus psychological content. This result, even if unexpected, is in line with previous findings in middle childhood (Bambini et al., 2024; Del Sette, Ronchi, Bambini, & Lecce, 2021). In their study, Del Sette and colleagues used the children's version of the PMM (12 items), and the confirmatory factor analysis revealed a unidimensional structure for accuracy, with physical and mental metaphors loading onto a single underlying latent variable. This suggests that articulating the meaning of a metaphor matures into a single ability, with similar patterns for different types of metaphor.

This study has some limitations that need to be acknowledged. First, we did not measure those cognitive skills that have been found to play a role in metaphor processing, such as working memory and executive control (Columbus et al., 2015; Domaneschi & Di Paola, 2019).^2^ However, recent studies pointed out that ToM may contribute more than EF to some pragmatic phenomena (Fairchild & Papafragou, 2021). Future works are needed to clarify the interplay between EF, general cognitive abilities, and age‐related differences in ToM in explaining metaphor skills—and pragmatics more broadly—in healthy aging (Bambini et al., 2021). A second caveat is related to the study design, which is correlational in nature. This means that no causal relationships could be inferred from our results. However, our models are based on robust theoretical frameworks and consistent empirical evidence, hence the current results can be considered a reliable first step in analyzing the mechanisms underlying age‐related differences in metaphor skills. Finally, our sample included more women than men, an imbalance that is common in aging research. Based on previous studies on ToM and metaphor skills (Daniluk & Borkowska, 2020; Di Tella, Miti, Ardito, & Adenzato, 2020; McDonald & Kanske, 2023), we did not expect gender to play a role in the pattern of associations tested, hence we did not include it in the SEM model. However, future work should seek to recruit a sample that is more representative of the aging population, to strengthen the generalizability of the results.

Notwithstanding these limitations, this study adopted an innovative approach and presents quite straightforward findings with relevant theoretical and practical implications. At the theoretical level, the study contributes to the lively debate on the interplay between pragmatics and social cognition (Andrés‐Roqueta & Katsos, 2020; Bosco et al., 2018; Mazzarella & Noveck, 2021). Focusing on metaphor comprehension as a part of pragmatic competence, our findings support the view of a link between pragmatics and social cognition, in line with theoretical claims (Sperber & Wilson, 2002), as well as developmental, clinical, and evolutionary evidence (Benítez‐Burraco, Ferretti, & Progovac, 2021; Champagne‐Lavau & Stip, 2010; Lecce et al., 2019). The present findings also show that the link between the pragmatic and social cognition domains should be intended as specific, with different aspects of ToM being differently engaged in different aspects of metaphor. Furthermore, our findings are particularly intriguing when considered from a lifespan perspective. Previous evidence indicated that ToM is associated with pragmatics in childhood, but this link seems to loosen during preadolescence and adulthood (Bosco, Parola, Sacco, Zettin, & Angeleri, 2017; Cappelli, Noccetti, Arcara, & Bambini, 2018; Lecce et al., 2019). Here, we found significant associations between the two domains in a middle‐to‐older adulthood sample, in harmony with evidence reported in adult clinical samples (Champagne‐Lavau & Stip, 2010; Cummings, 2013; Martin & McDonald, 2003). Besides its theoretical relevance, this study contributes to describing the linguistic profile of older adults, pointing to difficulty in metaphor as a possible “marker” to consider also for the description of pathological profiles, in line with recent research trends (Ivanova et al., 2022). In particular, a recent systematic review showed that impaired figurative language precedes impairment in literal language in people with dementia and, thus, is more sensitive than standard tests for the purpose of early detection of cognitive decline (Chakrabarty, Klooster, Biswas, & Chatterjee, 2023). More broadly, pragmatics has been shown to be a domain particularly susceptible to clinical worsening in neurodegenerative diseases, highlighting the importance of its assessment (Lago et al., 2022).

At the applied level, the present results confirmed that verbal communication difficulties are to be expected in older cohorts, and we believe that they deserve special attention due to their potential impact on social functioning (Daniluk & Borkowska, 2020; Palmer et al., 2019). Research on clinical populations consistently indicated that impairments in pragmatics negatively affect especially social relationships (Agostoni et al., 2021; Cummings, 2016), with metaphor being a particular predictive factor in some studies (Adamczyk et al., 2016; Bambini et al., 2020). While future studies are needed to provide direct evidence of the link between pragmatics and social functioning in healthy adulthood and aging, we can expect the link between these domains to hold also in the later stages of life. Given that the use of metaphors increases intimacy in social relationships (Bowes & Katz, 2015), difficulties in metaphor understanding experienced with increasing age might lead to less intimate and less satisfying interpersonal exchanges. Furthermore, as metaphors are a ubiquitous phenomenon in daily life (Steen, Dorst, Herrmann, Kaal, & Krennmayr, 2010), acknowledging age‐related difficulties in metaphor skills and examining the cognitive mechanisms underpinning this decline is key to designing intervention programs aimed at fostering pragmatics and communication more generally (Bambini et al., 2020). Pragmatics and communication training programs in turn can be key to promoting well‐being and a sense of belonging, especially for older adults (Chen, Yang, & Feng, 2019). The message that such programs can draw from our findings and the complex interplay of metaphor skills and ToM facets is that a joint intervention targeting both pragmatics and sociocognitive aspects might produce the greatest benefits.

## Supporting information



Supplementary material

## Notes

1 The Interpretation score closely mirrors the Intentionality score of the Animated Triangle task, where an ordinal scale is used to measure the degree of mental state attribution, from absence of mental state language to elaborate the use of mental state language, independently of accuracy (Castelli, 2002). In particular, the Interpretation score is equally applied to physical and mental metaphors, to capture the use of mental state attributions in explaining the metaphor independently on the accuracy in understanding the intended meaning. Consequently, a participant can score high in Interpretation even when the Accuracy is low, as in the case of “Lifeguards are lizards” explained as “They love to swim” or as “They are cunning and fool others.” Note that mental state terms might be inherent to the topic of the metaphor, as in the former example, or might be associated with the vehicle, as in the latter example, to both or to neither of them (see the Supplementary Material, Section A). For all aspects above, a possible better label for this score could be “mental interpretation,” as it is strictly related to mentalization degrees. However, we stick to “interpretation” for consistency with previous work (Del Sette, Bambini, Bischetti, & Lecce, 2020; Lecce, Ronchi, Del Sette, Bischetti, & Bambini, 2019).

2 Although we did not take specific cognitive measures, the assessment included the MMSE as a global index of cognitive functioning and we used it as an exclusion criterion. We did not include MMSE in the SEM model, but for exploratory purposes, we ran a correlation analysis using it as a rough index of cognitive functioning. Results showed that MMSE was moderately related to PMM accuracy score (*r* = .27, *p* < .001), supporting the idea that cognitive skills play a general role in metaphor understanding and pragmatic skills more broadly. However, MMSE was not related to PMM interpretation score (*r* = .13, *p* = .076). Interestingly, MMSE was not related to ToM either, neither accuracy (*r* = .06, *p* = .399) nor intentionality (*r* = .05, *p* = .474).
