# Supplementary material for: Aging and the Division of Labor of Theory of Mind Skills in Metaphor Comprehension
Source: Top Cogn Sci. 2025 Feb 17;18(4):e12785. doi: 10.1111/tops.12785 (PMC13740943; doi:10.1111/tops.12785)
Supplement: Supplementary file 1 [file TOPS-18-0-s001.pdf]

Aging and the division of labor of Theory of Mind skills in metaphor comprehension

Supplementary material

Section A. Physical and Mental Metaphors task (PMM)

The Physical and Mental Metaphors task (PMM) has been employed in childhood, adulthood, and aging, both in the oral and written form (Bambini et al., 2020; Canal et al., 2022; Del Sette et al., 2021; Lecce et al., 2019). The list of the metaphors included in the PMM used in the current study is reported in Table S1. Further details on the psycholinguistic characteristics of the items in the PMM are provided in the Supplementary material of the article "How to improve social communication in aging: Pragmatic and cognitive interventions" (Bambini et al., 2020).

Participants were instructed as follows: *"In questo compito troverà una serie di frasi contenenti espressioni come 'Quei ragazzi sono due gocce d'acqua'. Lei dovrà leggere le frasi e poi spiegare per iscritto che cosa significano. Cerchi di usare almeno 5 o 6 parole. Facciamo un esempio: lei leggerà la frase 'Quei ragazzi sono due gocce d'acqua'. Nelle righe sotto, scriverà che cosa significa, ad esempio 'Quei ragazzi si somigliano molto, hanno lo stesso aspetto'" (English translation: "In this task you will read some sentences presenting expressions such as "The two boys are water drops". Please, read each sentence and then write down what it means. Try to use at least five or six words. Let's see an example. You read the sentence "The two boys are water drops" and in the lines below you write what it means, such as "The two boys are very similar, they share their physical appearance").* Participants were randomly assigned to one among three lists. Each list differed in the order in which the 14 metaphors were presented.

Table S2 reports scoring examples. If a participant gave more than one explanation, we considered the one that obtained the highest score.

Finally, Table S3 reports the results of the salience analysis used to establish the validity of the scoring schema for the accuracy score. The accuracy score is based on the theoretical assumption that the meaning of nominal metaphors can be fully grasped, regardless of context, on the basis of salience, and can be described as the one that is conceptually salient, on the basis of conventionality, frequency/familiarity, and prototypicality (Del Sette et al., 2020). To establish salient meanings of the PMM metaphors, 76 university students (mean age = 22.08, *SD* = 1.52, mean years of education = 15, *SD* = 0.76) were presented with the PMM metaphors and were asked to verbalize the meaning of the metaphors. The responses were clustered in features, by collapsing answers with overlapping meanings and synonyms. For each metaphor, we evaluated whether the most salient features (i.e., the features that add up to at least the 50 % of the total number of features) were consistent with the answers receiving a score of 2 according to the PMM scoring schema. Results confirmed that the answers that were coded 2 points based on the scoring manual were indeed those indicating the features identified by the salience analysis.

Table S1. List of metaphors in the PMM task

**Physical set**		
Code	***Italian***	***English***
PMM_Phys_2	Certe ragazze sono spighe.	Some girls are grain spikes.
PMM_Phys_4	Quella sposa è una nuvola.	That bride is a cloud.
PMM_Phys_6	I vecchi sono tartarughe.	Old people are turtles
PMM_Phys_7	Quel pugile è un panda.	That boxer is a panda.
PMM_Phys_9	Certi cantanti sono usignoli.	Some singers are nightingales.
PMM_Phys_11	I bagnini sono lucertole.	Lifeguards are lizards.
PMM_Phys_13	I neonati sono batuffoli.	Newborns are cotton balls.
**Mental set**		
Code	***Italian***	***English***
PMM_Ment_1	Gli adolescenti sono pendoli.	Adolescents are pendulums.
PMM_Ment_3	I figli sono stampelle.	Children are crutches.
PMM_Ment_5	Certi amici sono ancore.	Some friends are anchors.
PMM_Ment_8	I filosofi sono aeroplani.	Philosophers are airplanes.
PMM_Ment_10	I nonni sono pilastri.	Grandparents are pillars.
PMM_Ment_12	Certi politici sono pavoni.	Some politicians are peacocks.
PMM_Ment_14	Certi professori sono lanterne.	Some professors are lanterns.

Table S2. Scoring examples for the PMM task

Metaphor	Scoring			
Mental				
Adolescents are pendulums	Cambiano idea ogni giorno.<br>*They change their opinions every day.*<br><br>Accuracy = 2<br>Interpretation = 3	Vanno avanti e indietro.<br>*They move back and forth.*<br><br>Accuracy = 1<br>Interpretation = 1	Cambiano bandiera.*<br>*They change their flag.*<br><br>Accuracy = 1<br>Interpretation = 2	Sono pieni di sogni e speranze.<br>*They are full of dreams and hopes.*<br><br>Accuracy = 0<br>Interpretation = 3
Physical				
Lifeguards are lizards	Stanno sempre sotto il sole.<br>*They spend all their time under the sun.*<br><br>Accuracy = 2<br>Interpretation = 1	Amano il sole.<br>*They love the sun.*<br><br>Accuracy = 2<br>Interpretation = 3	Nuotano tutto il giorno.<br>*They swim all day long.*<br><br>Accuracy = 0<br>Interpretation = 1	Amano nuotare.<br>*They love to swim.*<br><br>Accuracy = 0<br>Interpretation = 3

Note. Italian responses are reported (English translation in italics).

*This is an Italian idiomatic expression meaning time-serving change of opinion/line-up.

Table S3. Salient feature(s) and respective frequency for each metaphor in the PMM task

**Metaphor**	**Salient feature(s)**	**Frequency**
Certe ragazze sono spighe<br>*Some girls are grain spikes*	magrezza<br>*thinness*	42.86%
	altezza<br>*height*	19.05%
Quella sposa è una nuvola<br>*That bride is a cloud*	vestito bianco<br>*white dress*	46.67%
	vaporosità<br>*fluffiness*	22.22%
I vecchi sono tartarughe<br>*Old people are turtles*	lentezza<br>*slowness*	30.61%
	saggezza<br>*wisdom*	22.45%
Quel pugile è un panda<br>*That boxer is a panda*	livido<br>*bruise*	59.57%
Certi cantanti sono usignoli<br>*Some singers are nightingales*	voce<br>*voice*	81.40%
I bagnini sono lucertole<br>*Lifeguards are lizards*	stare al sole<br>*lying in the sun*	50.00%
I neonati sono batuffoli<br>*Newborns are cotton balls*	morbidezza<br>*softness*	28.30%
	tenerezza<br>*tenderness*	18.87%
	piccolezza<br>*smallness*	15.09%
Gli adolescenti sono pendoli<br>*Adolescents are pendulums*	instabilità<br>*instability*	50.00%
I figli sono stampelle<br>*Children are crutches*	supporto<br>*support*	45.28%
	aiuto<br>*help*	26.42%
Certi amici sono ancore<br>*Some friends are anchors*	riferimento<br>*reference*	19.70%
	affidabilità<br>*reliability*	18.18%
	supporto<br>*support*	15.15%
I filosofi sono aeroplani<br>*Philosophers are airplanes*	elevatezza<br>*elevation (of thought)*	39.58%
	pensiero<br>*thinking*	27.08%
I nonni sono pilastri<br>*Grandparents are pillars*	supporto<br>*support*	31.37%
	affidabilità<br>*reliability*	13.73%
	importanza<br>*importance*	9.80%
Certi politici sono pavoni<br>*Some politicians are peacocks*	atteggiarsi<br>*aptitude*	25.45%
	vanità<br>*vanity*	23.64%
	apparenza<br>*appereance*	18.18%
Certi professori sono lanterne<br>*Some professors are lanterns*	illuminare<br>*illuminating*	45.83%
	guida<br>*guide*	12.50%

Note. The salience frequency in the last column was calculated as the number of occurrences of that feature divided by the total number of answers for each metaphor.

Section B. Measurement models for Physical and Mental Metaphors (PMM), Strange Stories (SS), and Animated Social Triangles (AST) tasks

In all the measurement models the latent factor variance was fixed to 1 to scale the latent variables, allowing the factor loadings to be freely estimated. The mean- and variance-adjusted weighted least squares (WLSMV) estimator was used in estimating the measurement models for PMM accuracy, PMM interpretation, and SS, as these tasks had categorical items. ML estimator was used for the AST task.

Table S4. Fit indices and cut-offs used to assess model fit

Index	*Cut-off and interpretation*	*Reference*
χ^2 goodness of fit statistic	**Excellent fit:**<br>non-significant<br>χ^2/df ratio < 2	Carmines & McIver, 1981;<br>Kline, 2011
CFI (Bentler Comparative Fit Index)	**Excellent fit:**<br>CFI ≥ 0.95<br>**Adequate fit:**<br>CFI ≥ 0.90	Brown, 2015
TLI (Tucker-Lewis index)	**Excellent fit:**<br>TLI ≥ 0.95<br>**Adequate fit:**<br>TLI ≥ 0.90	Brown, 2015
RMSEA (Root Mean Square Error of Approximation)	**Excellent fit:**<br>RMSEA ≤ .05<br>90% CI lower limit ≤ .05<br>90% CI upper limit ≤ .10<br>**Adequate fit:**<br>RMSEA ≤ .08	Kline, 2011

PMM

We examined the PMM latent structure testing the fit of a two-factor model, in which mental and physical metaphors were considered as separate factors. Accuracy and interpretation were analyzed separately.

For accuracy, the two-factor model with seven items for each latent variable, namely Mental metaphors and Physical metaphors, showed good model fit, CFI = 0.95, TLI = 0.94,

RMSEA = .05, 90% CI [.04, .07], $\chi^2(76) = 121.42$, $p < .001$, χ^2/df ratio = 1.60. However, when inspecting parameter estimates, item 2 (loading onto the Physical metaphors latent factor) showed a non-significant amount of explained variance, $R^2 = .05$, $p = .162$. Therefore, we decided to remove this item from the PMM task in all the subsequent analyses. The measurement model without item 2 showed good fit to the data, $\chi^2(64) = 105.56$, $p <.001$, χ^2/df ratio = 1.65, CFI = 0.95, TLI = 0.94, RMSEA = .06, 90% CI [.04, .08]. As the correlation between the two latent factors obtained for accuracy (Mental metaphors and Physical metaphors) was very high, $r = .93$, we tested the fit of a unidimensional latent structure, with all 13 items loading onto a single latent variable. The unidimensional model showed good fit to the data, $\chi^2(65) = 107.16$, $p < .001$, χ^2/df ratio = 1.65, CFI = 0.95, TLI = 0.94, RMSEA = .06, 90% CI [.04, .08]. Finally, we compared the original two-factor model to the nested one-factor model. Model comparison was performed using the χ^2 difference test (Muthén & Muthén, 2012). Results showed nonsignificant deterioration in model fit moving from the two-factor model to the more parsimonious constrained model (i.e., the model with more degree of freedom), $\Delta\chi^2(1) = 1.94$, $p = .164$, hence supporting the one-factor solution. Results for the final, one-factor model are reported in Table S4.

Regarding the interpretation score, neither the two-factor ($\chi^2(64) = 107.89$, $p < .001$, χ^2/df ratio = 1.69, CFI = 0.87, TLI = 0.85), nor the one-factor model ($\chi^2(65) = 109.18$, $p < .001$, χ^2/df ratio = 1.68, CFI = 0.87, TLI = 0.85) showed satisfying fit to the data.

ToM

With respect to the SS task, results indicated excellent model fit, $\chi^2(5) = 2.38$, $p = .795$, χ^2/df ratio = 0.48, CFI = 1.00, TLI = 1.05, RMSEA = .00, 90% CI [.00, .06]. All estimated parameters significantly loaded onto a single latent factor, ps < .001, R^2s ≥ .21.

Regarding the AST task, as the model was just-identified, goodness of fit evaluation did not apply (Brown, 2015). We however inspected estimated parameters and found satisfying results, with all the indicators adequately loading onto the single latent factor, *p*s < .001.

Table S5. Results of the unifactorial model for the PMM accuracy (with 13 items) and parcels' creation

Item	*Standardized estimate (ascending order)*	*Standard error*	*Parcel*
MET_6Phy_acc	0.471	0.101	M1
MET_1Men_acc	0.544	0.060	M2
MET_5Men_acc	0.556	0.058	M3
MET_10Men_acc	0.560	0.063	M3
MET_8Men_acc	0.577	0.059	M2
MET_14Men_acc	0.583	0.065	M1
MET_7Phy_acc	0.586	0.066	M1
MET_4Phy_acc	0.589	0.062	M1
MET_3Men_acc	0.649	0.069	M2
MET_11Phy_acc	0.705	0.069	M3
MET_12Men_acc	0.726	0.067	M3
MET_13Phy_acc	0.741	0.064	M2
MET_9Phy_acc	0.919	0.083	M1

Note. Parcel creation was done following Little and colleagues' recommendations (2002). Specifically, we adopted the *balancing approach*, grouping the items based on their factor loadings (Little et al., 2013, p. 15).

References

Bambini, V., Tonini, E., Ceccato, I., Lecce, S., Marocchini, E., & Cavallini, E. (2020). How to improve social communication in aging: Pragmatic and cognitive interventions. *Brain and Language*, *211*, 104864. https://doi.org/10.1016/j.bandl.2020.104864

Brown, T. (2015). *Confirmatory factor analysis for applied research*. Guilford Press. https://doi.org/10.1680/geot.8.B.012

Canal, P., Bischetti, L., Bertini, C., Ricci, I., Lecce, S., & Bambini, V. (2022). N400 differences between physical and mental metaphors: The role of Theories of Mind. *Brain and Cognition*, *161*, 105879. https://doi.org/10.1016/j.bandc.2022.105879

Carmines, E. G., & McIver, J. P. (1981). Analyzing models with unobserved variables: Analysis of covariance structures. In G. W. Bohrnstedt & E. F. Borgatta (Eds.), *Social measurement: Current issues* (pp. 66–115). SAGE Publications.

Del Sette, P., Bambini, V., Bischetti, L., & Lecce, S. (2020). Longitudinal associations between theory of mind and metaphor understanding during middle childhood. *Cognitive Development*, *56*, 100958. https://doi.org/10.1016/j.cogdev.2020.100958

Del Sette, P., Ronchi, L., Bambini, V., & Lecce, S. (2021). Longitudinal associations between metaphor understanding and peer relationships in middle childhood. *Infant and Child Development*, *30*(4). https://doi.org/10.1002/icd.2232

Kline, R. B. (2011). *Principles and practice of structural equation modeling* (3rd ed.). Guilford Press.

Lecce, S., Ronchi, L., Del Sette, P., Bischetti, L., & Bambini, V. (2019). Interpreting physical and mental metaphors: Is Theory of Mind associated with pragmatics in middle childhood? *Journal of Child Language*, *46*(2), 393–407. https://doi.org/10.1017/S030500091800048X

Little, T. D., Cunningham, W. A., Shahar, G., & Widaman, K. F. (2002). To parcel or not to

parcel: Exploring the question, weighing the merits. *Structural Equation Modeling*, *9*(2), 151–173. https://doi.org/10.1207/S15328007SEM0902_1

Little, T. D., Rhemtulla, M., Gibson, K., & Schoemann, A. M. (2013). Why the items versus parcels controversy needn't be one. *Psychological Methods*, *18*(3), 285–300. https://doi.org/10.1037/a0033266

Muthén, L. K., & Muthén, B. O. (2012). *Mplus User's Guide* (7th ed.).
